# A Concise Review of Ketogenic Dietary Interventions in the Management of Rare Diseases

**DOI:** 10.1155/2021/6685581

**Published:** 2021-02-15

**Authors:** D. Hettiarachchi, K. Lakmal, V. H. W. Dissanayake

**Affiliations:** ^1^Department of Anatomy, Faculty of Medicine, University of Colombo, Colombo, Sri Lanka; ^2^Human Genetics Unit, Faculty of Medicine, University of Colombo, Colombo, Sri Lanka

## Abstract

Dietary interventions are now being used as an adjunct therapy in the treatment of rare diseases. One such method is the high-fat, moderate-protein, and very low-carbohydrate diet which produces ketosis and therefore called the ketogenic diet. Some of the more common conditions that are treated with this method are pharmacoresistant epilepsy, infantile spasms, glycogen storage diseases, and other forms of rare metabolic disturbances. With this review, we look at different uses of the ketogenic diet in treating rare diseases and the recommendations based on current evidence.

## 1. Introduction

There are many obstacles when treating rare diseases as they may often depict only the tip of the iceberg, which represents some varied genetic and phenotypic characteristics. Certain dietary modifications such as the ketogenic diet and the modified Atkins diet (MAD) have been tried in a plethora of rare diseases ranging from epilepsy, infantile spasms, and glycogen storage diseases to other forms of rare metabolic disturbances [1–3]. In monogenic rare diseases, a continuum of therapeutic strategies can be employed, and medical nutrition therapy is one such option. With the expansion of our understanding of the affected metabolic pathways, we can now tailor these therapies on a personalized basis [4]. A ketogenic diet is one that is typically high in fat, with moderate protein and a very low-carbohydrate levels, or it can also be high in protein and lower in fat and carbohydrate levels, with the end result of producing ketosis. This produces a form of starvation and forces the body to deviate from its traditional fuel source glucose to primarily use fats [5]. The key to a ketogenic diet is limited carbohydrate intake to a total daily amount of 10 to 50 grams or 5% to 10% of total caloric intake which causes fat to be converted to various ketone bodies for cellular metabolism (e.g., acetoacetate, beta-hydroxybutyrate, and acetone) [6, 7]. Certain ketogenic dietary protocols use medium-chain triglycerides (MCT) to help boost ketone production as they are more rapidly broken down into ketones and energy [8]. This form of dietary modifications was documented since 1925 to treat drug-resistant epilepsy [9, 10]. However, a prolonged ketogenic diet is not endured well as there are several drawbacks such as retardation of growth in children and gastrointestinal side effects [11]. The aim of this review is to look at the different uses of a ketogenic diet in treating rare diseases and to make recommendations based on current evidence.

## 2. Pharmacoresistant Epilepsy, Infantile Spasms, and Tuberous Sclerosis

It is estimated that 30% of epilepsy patients are pharmacoresistant. In this group of patients, a ketogenic diet (KD) has offered some promising results [12]. In experimental cell culture models such as in rat hippocampal neuronal cells, the 3 ketone bodies, namely, acetone, acetoacetic acid, and *β*-hydroxybutyric acid, inhibit the opening of the acid-sensing ion channels through undermining their currents and, as a result, inhibit their function [11]. From the literature review done in MEDLINE, Google Scholar, and Cochrane library, six randomized control trials [13–18] have assessed the efficacy and safety of different KD regimes in patients with intractable childhood epilepsy. All 6 studies showed beneficial effects, especially in reducing the baseline seizure frequency. Findings from these studies are summarized in Table 1. The adverse side effects of KD included digestive problems such as diarrhea, constipation, vomiting, and issues related to lipid metabolism such as hyperlipidemia, hypercalciuria, and weight loss. The studies conducted thus far had the limitation of small sample size in the pediatric age group [19]. There were 5 randomized control trials [20–24] which were conducted to compare the efficacy between different KD regimes. These studies have compared different KD regimes by taking into account the efficacy and adverse effects. Findings from these studies are summarized in Table 2. A majority of these studies (*n* = 4) have not shown significant differences in efficacy; however, different regimes had different side effect profiles.

Infantile spasms (IS) are a form of epilepsies that occur in infancy and have a characteristic EEG pattern (hypsarrhythmia) accompanied sometimes with intellectual disability and neurodevelopmental regression [1]. Ketogenic diet has been tried with success; however, it has been looked at with reluctance by pediatricians owing to the age of patients and unknown side effects. In a larger study where 104 infants were started on KD after treatment failure with corticosteroids and vigabatrin, it was reported that 64% saw an improvement in spasms 6 months after starting treatment and 77% saw improvements following 1-2 years, and adverse effects were reported only in 33%. However, in 6%, there was diminished linear growth. They also observed that the older the onset of IS and fewer the anticonvulsant use, the greater the likelihood of improvement of spasms on a KD [25].

In the tuberous sclerosis complex (TSC), a condition frequently associated with epilepsy and infantile spasms; a KD has been tried with success. In a study spanning 5 years conducted at Johns Hopkins Hospital and Massachusetts General Hospital, 12 children between the ages of 8 and 18 years were started on a KD. Among them, 92% (*n* = 11) showed a significant (>50%) improvement in the initial 6 months itself [26]. In another study, 21 out of 31 patients showed a significant reduction in seizures (>50%) in the initial 3 months of initiating a KD [27]. Both studies have concluded that KD appeared to be effective as a therapeutic intervention in intractable pediatric epilepsy in TSC. However, its long-term efficacy needs further evaluation.

## 3. Inherited Metabolic Disorders

### 3.1. Glycogen Storage Diseases (III and V)

Glycogen storage disease type III (GSD III) is an autosomal recessive disease caused by the deficiency of the glycogen debranching enzyme, encoded by the AGL gene. It is characterized by variable liver, cardiac muscle, and skeletal muscle involvement. GSD IIIa is the most common subtype present in about 85% of affected individuals; it manifests with liver and muscle involvement. GSD IIIb, with liver involvement only, comprises about 15% of all GSD III [28]. Currently, the treatment to limit glycogen storage is the diet. High-carbohydrate diet prevents fasting hypoglycaemia but increases glycogen storage and does not slow the progression of cardiac and muscular manifestation [29]. Several case reports and case series report beneficial effects on the muscle disease component of GSD IIIa with KD (study details are summarized in Table 3).

GSD V disease is caused by genetic defects of the muscle-specific isozyme of glycogen phosphorylase, which blocks ATP formation from glycogen in skeletal muscles [34]. Patients with this rare disease present with exercise intolerance due to blocked glycogen breakdown in skeletal muscles. Introducing alternative fuel substrates, such as ketone bodies (by providing a ketogenic diet), could potentially alleviate muscle symptoms. The summary of case reports relevant to the management of GSD V with KD is given in Table 3.

## 4. Mitochondrial Disorders

### 4.1. Pyruvate Dehydrogenase Complex Deficiency

The pyruvate dehydrogenase complex (PDHc) is a member of a family of multienzyme complexes that provides the link between glycolysis and the tricarboxylic acid (TCA) cycle by catalyzing the decarboxylation of various 2-oxoacid substrates to their corresponding acyl-CoA derivatives, NADH, and CO_2_ [37]. PDHc deficiency is a metabolic disorder commonly associated with lactic acidosis and progressive neurological and neuromuscular degeneration [38]. In 1976, Falk et al. showed that KD would be beneficial in PDHc deficiency [39, 40]. Since then, several case reports report the efficacy of the KD in the management of this disease (Table 4).

### 4.2. Mitochondrial DNA Depletion Syndromes (MDS)

Mitochondrial DNA depletion syndromes are characterized by a severe, tissue-specific decrease in the mitochondrial DNA (mtDNA) copy number with resulting organ failure. Polymerase gamma (POLG) is one of the enzymes catalyzing mtDNA replication. MDS are phenotypically heterogeneous and usually classified as myopathic, encephalomyopathic, hepatocerebral, or neurogastrointestinal.

## 5. Conclusion

The KD is an efficacious therapy for pharmacoresistant epilepsy in children, and it should be considered strongly after failure of traditional treatment. It has several gastrointestinal side effects which can be modified with different ketogenic diet regimes. Before prescribing such dietary modifications, it is vital that parents or the caregivers in younger children are able to comprehend and carry out such a nutritional plan intelligently.

The main indications to start with a KD in GSD III patients were cardiomyopathy, skeletal myopathy, or a combination of both, and the KD had good beneficial outcomes according to the existing case reports. Treatment of KD in mitochondrial diseases also showed promising results in improvement of the clinical picture, but for these diseases, evidence is limited as these diseases are rare, and available evidence is in the form of case reports or case series. Furthermore, carefully designed systematic studies are warranted in patients with rare metabolic diseases to address issues of dietary compliance and the effect of both standard and ketogenic diets on short- and long-term outcomes.

## Figures and Tables

**Table 1:** Randomized control trials describing efficacy and safety of ketogenic diet compared with other \treatment modalities.

	Author	Year	Country	Study population	Type of KD	Interventions	Sample size	Results	Adverse effects	Cost effectiveness
1	Dressler et al.	2019	Austria	Infants with West syndrome	Without fasting and fluid restriction, 1 : 1 fat : nonfat, and individually increased to 3 : 1(ratio was limited when beta‐hydroxybutyrate levels reached >5 mmol L).	Group 1: ketogenic diet (KD) Group 2: standard ACTH treatment	Group 1: *n* = 16 Group 2: *n* = 16	Electroclinical remission at 28 days was nonsignificant (62% (KD) vs. 69% (ACTH)) Without prior vigabatrin treatment, remission at day 28 was 47% (KD) and 80% (ACTH, *p*=0.02) Relapse rate 16% (KD) and 43% (ACTH) (*p*=0.09) Age‐appropriate psychomotor development and adaptive behaviour were similar.	Adverse effects needing acute medical intervention: 30% (KD) vs. 94% (ACTH) (*p* < 0.001)	N/A

2	Wijnen et al.	2017	Netherlands	Children and adolescents with intractable epilepsy	Classical ketogenic diet, the medium-chain triglyceride (MCT) diet, or a mixture of both diets.	Group 1: ketogenic diet Group 2: care as usual (CAU) with AEDs	Group 1: *n* = 26 Group 2: *n* = 22	At 16 months, 35% of the KD participants had a seizure reduction ≥ 50% from baseline, compared with 18% of the CAU participants; 46.2% of the KD group reported a decrease in the severity of their worst seizure compared to 32% of the CAU group.	At 4-month follow-up, the KD group showed significantly more gastrointestinal problems compared to the CAU group (*p* < 0.001). At 16 months, the KD group reported fewer problems compared to CAU (*p*=0.032).	Mean costs per patient in the CAU group were 53,367 euros compared to 61,019 euros per patient in the KD group

3	Lambrechts et al.	2016	Netherlands	Refractory epilepsy patients aged 1–18 years	Most frequent was the MCT diet. When only tube feeding was given, a liquid form of the classical KD was used.	Group 1: ketogenic diet Group 2: care as usual (continued taking usual AEDs)	Group 1: *n* = 26 Group 2: *n* = 22	13 patients (50%) treated with the KD and four patients (18.2%) of the CAU group were responders. Mean seizure frequency at 4 months compared to baseline was significantly lower (*p*=0.024) in the KD group (56%) (95% CI: 36–76) than in the CAU group (99%) (95% CI: 65–133%). Twice as many patients in the KD group had a relevant decrease in the seizure severity score (*p*=0.070).	Patients treated with the KD had a significantly higher score for gastrointestinal symptoms (*p*=0.021) without an increase in the total score of side effects.	N/A

4	Ijff et al.	2016	Netherlands	Refractory epilepsy patients aged 1–18 years	N/A	Group 1: KD with AEDs Group 2: only AED (CAU)	Group 1: *n* = 28 Group 2: *n* = 22	The KD group showed lower levels of anxious and mood-disturbed behaviour and was rated as more productive. Cognitive test results showed an improvement of activation in the KD group.	N/A	N/A

5	Kim et al.	2015	Korea	Intractable childhood epilepsy	4 : 1 lipid to nonlipid ratio and nonfasting initiation protocol.	Group 1: KD Group 2: modified Atkins diet (MAD)	Group 1: *n* = 51 Group 2: *n* = 53	KD group had a lower mean percentage of baseline seizures compared with the MAD group at 3 months (38.6% for KD; 47.9% for MAD) and 6 months (33.8% for KD; 44.6% for MAD), but the differences were not statistically significant (95% confidence interval (CI): 24.1–50.8; *p*=0.021 for 3 months; 95% CI: 17.8–46.1; *p*=0.255 for 6 months). The rate of seizure freedom at 3 months after diet therapy initiation was significantly higher (53% for KD; 20% for MAD;*p*=0.047) in patients aged 1-2 years.	The MAD had advantages with respect to better tolerability and fewer serious side effects.	N/A

6	Neal et al.	2008	United Kingdom	Drug-resistant epilepsy children aged between 2 and 16 years	Classical diets were started at a 2 : 1 ratio (fat : protein and carbohydrate) and gradually increased to a 3 : 1 or 4 : 1 ratio over 1-2 weeks, as tolerated.	Group 1: KD Group 2: control group—care as usual (with AEDs)	Group 1: *n* = 54 Group 2: *n* = 49	After 3 months, the mean percentage of baseline seizures was significantly lower in the KD group than in the controls (62.0% vs. 136.9%, 75% decrease; 95% CI: 42.4–107.4; *p* < 0.0001). 28 children (38%) in the KD group had greater than 50% seizure reduction compared with four (6%) controls (*p* < 0.0001), and five children (7%) in the diet group had greater than 90% seizure reduction compared with no controls (*p*=0.582). No significant difference in the efficacy of the treatment between symptomatic generalised or symptomatic focal syndromes.	The most frequent side effects reported at a 3-month review were constipation, vomiting, lack of energy, and hunger.	N/A

KD: ketogenic diet; ACTH: adrenocorticotropin hormone; MCT: medium-chain triglyceride; CAU: care as usual; AEDs: antiepileptic drugs; N/A: not available.

**Table 2:** Efficacy and tolerability of different ketogenic diet regimens for children with intractable epilepsy in 5 randomized control trials.

	Author	Year	Study population	Sample size	Intervention of group 1	Intervention of group 2	Follow-up period	Efficacy of the diet	Adverse events
1	Bergqvist et al.	2005	Intractable epilepsy (1–14 years' age group)	Group1: *n* = 24 Group 2: *n* = 24	Fasting initiation of KD	Nonfasting gradual initiation of KD (ketogenic ratio from 1 : 1 to 4 : 1)	3 months	No significant difference	Less weight loss, fewer and less severe episodes of hypoglycaemia, and fewer treatments for acidosis and dehydration in group 2
2	Seo et al.	2007	Intractable childhood epilepsy	Group 1: *n* = 22 Group 2: *n* = 36	Diet with nonlipid : lipid ratio 4 : 1	Diet with nonlipid : lipid ratio 3 : 1	3 months	Higher in the 4 : 1 diet group	Less gastrointestinal symptoms in group 2
3	Neal et al.	2009	Childhood intractable epilepsy	Group 1: *n* = 61 Group 2: *n* = 64	Classic diet	Medium-chain triglyceride diet	12 months	No significant difference	Equivalent except increased reports of lack of energy and vomiting in group 1
4	Kang et al.	2011	Refractory infantile spasms	Group 1: *n* = 16 Group 2: *n* = 19	Short-term (8 months) KD	Long-term (>2 years) KD	More than 2 years	No significant difference	No growth disturbance and osteopenia in group 1
5	Raju et al.	2011	Childhood refractor epilepsy (6 months–5 years)	Group 1: *n* = 19 Group 2: *n* = 19	Diet with nonlipid : lipid ratio 4 : 1	Diet with nonlipid : lipid ratio 2.5 : 1	3 months	No significant difference	Less constipation, weight loss, and hospitalization for lower respiratory tract infections in group 2

KD: ketogenic diet.

**Table 3:** Summary of case report and case series found in the literature regarding use of KD in glycogen storage diseases.

Type of glycogen storage disease	Author	Year	Patient characteristics	Traditional treatment method	Dietary modification	Results
Type IIIa	Brambilla et al. [31]	2014	Two siblings (girl and boy), 7 and 5 years old, both with left ventricular hypertrophy	Frequent diurnal and nocturnal hyperproteic meals followed by orally administered uncooked corn starch	High-fat (60%), high-protein (25%), and low-carbohydrate (15%) diet.	Both patients showed a relevant reduction of the thickness of interventricular septum, left ventricle posterior wall, and an improvement of the outflow obstruction.
Type IIIa	Mayorandan et al. [1]	2014	2 boys (9 and 11 years)	Frequent feeds with carbohydrate-rich meals or continuous enteral feeding	A modified Atkins diet (10 g carbohydrate per day, protein, and fatty acids ad libitum) over a period of 32 and 26 months, respectively, in 9 years old and 11 years old.	Creatine kinase levels in blood dropped in response to Atkins diet. One patient suffered from severe cardiomyopathy which significantly improved under the diet. Apart from transient hypoglycaemia, no serious adverse effects were observed.
Type IIIa	Francini et al. [32]	2019	A 34-year-old male patient with hypertrophic cardiomyopathy	High-carbohydrate diet	Modified Atkins diet (MAD) providing up to 20 g carbohydrate per day which is roughly equivalent to a ratio of 1-2 : 1 of fat to protein plus carbohydrates.	After 12 months of treatment, ejection fraction raised from 30 to 45%, liver enzymes were reduced, and CK plasma level dropped from 568 to 327 U/l. Physical activity increased from about 1300 to 2800 steps per day, and health-related quality of life assessment ameliorated. An increase in the uric acid triglycerides plasma level was observed.
Type IIIa	Olgac et al. [33]	2020	6 patients, aged 3–31 years	Frequent feeds with high complex carbohydrates in small children and a low-carb-high-protein diet in older children and adults	Modified Atkins diet.	In all patients, transaminase levels dropped in response to MAD. Decrease in CK levels was detected in 5 out of 6 patients. Hypoglycemia was evident in 2 patients but was resolved by adding uncooked corn starch to diet.
Type IIIa	Marusic et al. [35]	2020	A 15-year-old girl with left ventricular obstructive hypertrophy	Frequent corn starch meals	Diet consisted of ketogenic ratios of 2.5 : 1; fats (5.2 g/kg/d) contributed 87% daily calories, proteins (1.6 g/kg/d) contributed 11%, and carbohydrates (0.3 g/kg/d) contributed 2%. Continuous ketosis was maintained for over 4 years.	Cardiac MRI was repeated after 16 and after 40 months, showing a normalization of left ventricular parameters, with a decrease of the total left ventricular mass index (LVMI) (from 58 g/m^2^ to 37 g/m^2^) without residual outflow obstruction. Finally, no hypoglycemic events were recorded while on KD.
Type V	Vorgerd et al. [30]	2002	A 55-year-old male	Carbohydrate-based diet	Creatine supplementation and ketogenic diet (increasing the fat content of his diet to 80% with 14% protein (1 g/kg/d) in total).	With a continuous one-year diet, his exercise tolerance was 3- to 10-fold increased, dependent of the endurance level. Maximum strength and activity duration also improved, and CK levels dropped from 5.300 U/l to 890 U/l on ketogenic diet.
Type V	Reason et al. [36]	2017	3 patients (a 54-year-old male, a 12-year-old female, and a 45-year-old female)	Care as usual	Low-carbohydrate ketogenic diet for 6 months.	Distinct improvement in activity and exercise tolerance was found. Plasma creatine kinase (CK) was significantly lowered in all three cases, with two patients exhibiting values within normal range. Overall, each patient described an improved quality of life.
Type V	Lokken et al. [37]	2019	8 patients	Care as usual	Participants were randomized to follow one of the three KD regimes for 3 weeks (#1: 65%/15%/20%; #2: 75%/15%/10%, or #3: 80%/15%/5%, fat/protein/carbohydrate).	Five patients reported subjective symptom relief. All diet regimes seemed to improve fatty acid oxidation rates and exercise capacity.

**Table 4:** Summary of case report and case series found in the literature regarding use of KD in mitochondrial disorders.

Mitochondrial disorder	Author	Year	Patient characteristics	Traditional treatment method	Dietary modification	Results
Pyruvate dehydrogenase complex deficiency	Falk et al. [40]	1976	Two brothers, aged 11 years 6 months and 2 years 3 months, with psychomotor and growth retardation, episodes of weakness, ataxia, ophthalmoplegia, and elevated levels of blood pyruvate	Standard glucose meal	Ketogenic diet.	Fall in blood pyruvate levels, a decrease in the frequency and severity of the episodes of neurological deterioration, an increased rate of growth and development in the younger brother, and increased strength and endurance in the older one.
	Sofou et al. [41]	2017	19 patients (3 boys and 16 girls)	Usual diet	Ketogenic diet (2.5 : 1 fat : carbohydrate plus protein) for a median of 2.9 years.	The treatment had a positive effect mainly in the areas of ataxia, sleep disturbance, speech/language development, social functioning, and frequency of hospitalizations. It was also safe—except in one patient who discontinued because of acute pancreatitis.
	Chida et al. [42]	2018	A 11-year-old girl	Medium-chain triglyceride (MCT) formula and a trace mineral supplemental drink.	Ketone formula was begun at 50 g/1,200 mL, four times per day; the amount was gradually increased to 76 g/1,200 mL, four times per day with a regular checkup of total ketone body level.	Seizure frequency markedly decreased, but the electroencephalogram findings, which included a spike-and-wave pattern arising from the bilateral occipital region and expanding throughout the region, did not markedly improve after the introduction of the KD.
	El-Gharbawy et al. [43]	2011	A 15-month-old boy	Breast feeding	Meals with a ketogenic ratio (KR) of 4 : 1 fat (grams) : protein plus carbohydrate (grams). Later, to improve compliance, the KR of meals was decreased to 3 : 1, and medium-chain triglyceride (MCT) oil was added.	Seizures and hyperventilation ceased.

Mitochondrial DNA depletion syndromes	Joshi et al. [44]	2009	A 31-month-old girl with Alpers-Huttenlocher syndrome	Usual diet	Ketogenic diet.	Improvement in clinically, and her electroencephalogram improved dramatically.
	Cardenas et al. [45]	2010	A 14-month-old baby girl with compound heterozygous polymerase gamma gene mutation	Usual diet	Ketogenic diet.	Reduction in seizure frequency.

## Data Availability

There are no additional data other than which are mentioned in the article already.
